# Glucagon-like peptide-1 agonists in cardiovascular diseases: a bibliometric analysis from inception to 2023

**DOI:** 10.1097/MS9.0000000000002592

**Published:** 2024-09-25

**Authors:** Abinash Mahapatro, Ali Bozorgi, Sri U.J. Obulareddy, Shika M. Jain, Rohan Reddy Korsapati, Aroon Kumar, Kristina Patel, Saman Soltani Moghadam, Arash Arya, Abdulhadi Jameel Alotaibi, Mohammad-Hossein Keivanlou, Soheil Hassanipour, Maryam Hasanpour, Ehsan Amini-Salehi

**Affiliations:** a Hi-Tech Medical College and Hospital, Rourkela, Odisha, India; b Tehran Heart Center, Tehran University of Medical Sciences, Tehran, Iran; c University of Arkansas, Little Rock, Arkansas, USA; d MVJ Medical College and Research Hospital, Bengaluru, India; e University of Toledo, Toledo, Ohio, USA; f Baylor College of Medicine, Houston, USA; g Shenyang North New Area, Shenyang, Liaoning Province, People’s Republic of China; h Gastrointestinal and Liver Diseases Research Center, Guilan University of Medical Sciences, Rasht, Iran; i Department of Internal Medicine III, Halle University Hospital, Halle (Saale), Germany; j Vision Colleges, Riyadh, Saudi Arabia

**Keywords:** bibliometric analysis, cardiovascular diseases, diabetes mellitus, glucagon-like peptide-1, trend

## Abstract

**Background:**

In recent years, glucagon-like peptide-1 (GLP-1) agonists have garnered increasing attention for their potential cardiovascular benefits beyond glycemic control in patients with diabetes. Understanding the research landscape surrounding GLP-1 agonists and cardiovascular diseases (CVDs) is crucial for informing clinical practice and guiding future research endeavors. This bibliometric analysis aimed to comprehensively assess the scholarly output and trends in this field, shedding light on the evolving landscape of GLP-1 agonists’ role in cardiovascular health.

**Methods:**

The publications concerning GLP-1 agonists in CVDs were gathered from the Web of Science Core Collection, and visualizations were created utilizing Excel 2019, Cite Space, and VOS viewer software.

**Results and Conclusion:**

Using bibliometric and visual methods, the research hotspots and trends regarding GLP-1 agonists in cardiovascular diseases were pinpointed. Additionally, a thriving interest in GLP-1 agonists research within cardiovascular medicine was observed, with a notable surge in publications from 2016 onwards. The analysis revealed that the United States and China are the leading contributors, accounting for over 50% of the total publications. The University of Copenhagen and the University of Toronto emerged as the most prolific institutions in this field. Co-citation analysis highlighted the influential role of landmark clinical trials, such as the LEADER, ELIXA, and EXSCEL. Keyword trend analysis identified the emergence of newer GLP-1 agonists, such as tirzepatide and semaglutide, as well as a growing focus on topics like ‘healthy obesity’ and chronic kidney disease. These findings suggest that the research landscape is evolving, with a focus on expanding the therapeutic applications of GLP-1 agonists beyond glycemic control. Overall, this bibliometric analysis provided insights into the current state and future directions of research on GLP-1 agonists and their impact on cardiovascular health, guiding future research endeavors, and informing clinical practice.

## Graphical abstract

**Figure:**
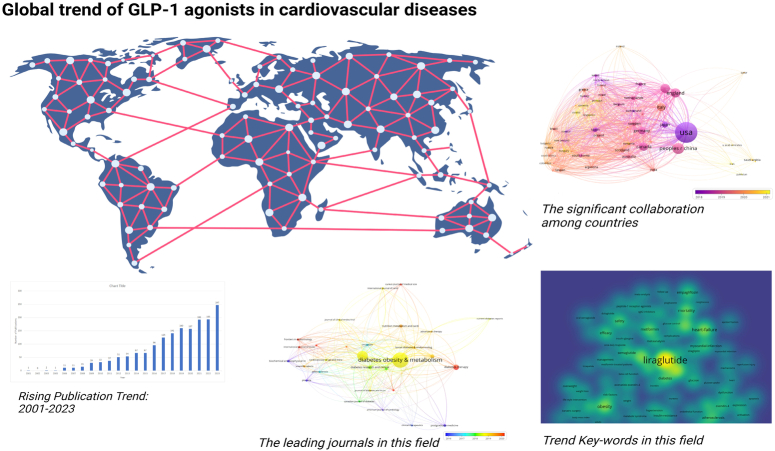

## Introduction

Highlights

- GLP-1 agonists are renowned for their potential role in the treatment of diabetes mellitus; however, they have also garnered attention for their cardiovascular benefits.
- To date, numerous studies have explored the potential of GLP-1 agonists in cardiovascular disorders. Nonetheless, there exists a need for a bibliometric analysis to comprehensively assess the scholarly output and trends in this field, thereby shedding light on the evolving landscape of GLP-1 agonists’ role in cardiovascular health.
- Our bibliometric study yielded compelling results, indicating an interest in GLP-1 agonists research within cardiovascular medicine. Additionally, recent studies have underscored emerging topics in GLP-1 research in CVDs, including investigations into new drugs such as tirzepatide and semaglutide, as well as explorations into heart failure with preserved ejection fraction (HFpEF) and kidney outcomes.

In the landscape of metabolic disorder treatments, glucagon-like peptide-1 (GLP-1) agonists have emerged as a significant class of therapeutic agents, offering a multifaceted approach in managing conditions such as type 2 diabetes mellitus (T2DM) and obesity^1–4^. These agents mimic the action of the endogenous hormone GLP-1, enhancing glucose-dependent insulin secretion, suppressing glucagon secretion, and slowing gastric emptying^5,6^. Beyond their glycemic benefits, GLP-1 agonists have garnered attention for their potential protective effects on the cardiovascular system and their role in the management of hepatic diseases, such as nonalcoholic fatty liver disease (NAFLD) and nonalcoholic steatohepatitis (NASH)^7–14^.

Cardiovascular diseases (CVDs) remain the leading cause of mortality worldwide, presenting a significant public health challenge^15–21^. The intricate relationship between metabolic disorders and cardiovascular health has been well-documented, with conditions like T2DM and obesity significantly increasing the risk of developing CVDs^22–28^. In this context, treatments that address metabolic dysfunctions while also offering cardiovascular protective effects are of paramount importance. Recent studies and clinical trials have highlighted the potential of GLP-1 agonists in reducing cardiovascular risk factors, making them an area of keen interest for researchers and clinicians alike^29–33^.

The increasing trend of utilizing GLP-1 agonists in the management of CVDs is reflective of a broader shift towards integrative therapeutic strategies that encompass a wide range of metabolic and cardiovascular benefits^34^. This trend underscores the need for comprehensive evaluations of the efficacy, safety, and mechanisms of action of these agents, particularly in populations with complex comorbidities such as hepatic diseases.

Bibliometric studies serve as a powerful tool in this evaluative process, providing insights into the structure, dynamics, and evolution of scientific research^35^. By systematically analyzing publication patterns, citation networks, and thematic evolutions within the scientific literature, bibliometric analyses can uncover the impact, trends, and gaps in the current body of knowledge. This methodological approach allows for a quantitative assessment of research activity and influence, facilitating a deeper understanding of the scientific landscape surrounding specific research areas^36–38^.

This study aimed to conduct a comprehensive bibliometric analysis of the literature surrounding GLP-1 agonists, with a specific focus on their application in managing CVDs. By mapping out the research trajectory, identifying key contributors, and assessing the thematic and methodological trends within this field, this study sought to elucidate the current state of knowledge and pinpoint areas where further research is needed.

## Methods

### Data collection

To carry out our research on the impact of GLP-1 agonists on cardiovascular diseases, we selected the Web of Science Core Collection as our principal database for inquiry, conducting our search on 19 February 2024. This database is distinguished by its comprehensive compilation of information, encompassing more than 12 000 reputable journals^39–41^. To maximize the search’s efficiency, we crafted an elaborate search strategy that integrated a variety of keywords, as detailed in Table S1 (Supplemental Digital Content 1, http://links.lww.com/MS9/A604).

We did not impose any restrictions regarding the timeframe for the studies considered. Initially, we identified 1968 articles. Subsequently, through the exclusion of conference proceedings, letters, editorials, book chapters, prepublication articles, and studies not relevant to our focus, we refined our selection to 1697 pertinent studies.

### Data analysis

All the relevant documents sourced from the Web of Science Core Collection were converted to Microsoft Excel 2019, and plain text formats and analyzed using VOS viewer and Cite Space. VOS viewer stands out as a powerful tool for scientometric network analysis, established by the Center for Science and Technology Studies at Leiden University, Netherlands. This software excels in providing visual insights and generating maps based on network data, facilitating an understanding of the relationships within academic literature. It is adept at constructing network diagrams that represent various academic entities such as publications, journals, authors, research institutions, countries, and keywords, connecting them through different types of links like cocitation, co-occurrence, citation, and bibliographic coupling.

VOSviewer offers three distinct types of visualization maps: network, overlay, and density visualizations, each serving a unique analytical purpose^42^. The foundational concept behind the software’s design is cooccurrence clustering, which signifies the relatedness of items within the network. This approach allows for the identification of correlations of varying strengths and directions. By analyzing the clustering of these relationships based on intensity and direction, one can discern distinct groups within the data. Although primarily utilized for bibliometric analyses, VOS viewer’s capabilities extend to the creation of a wide range of web data maps. Its most notable attribute is the generation of high-quality visual graphics, making it exceptionally suitable for large-scale academic and scientific visual analyses^36,43^.

Cite Space software, developed by Professor Chen Chaomei at Drexel University, leverages Java programming language to offer citation visualization analysis grounded in scientometrics and data visualization principles. This innovative tool illuminates the structure, dynamics, and distribution patterns of scientific knowledge through advanced data mining techniques, comprehensive information analysis, and the generation of detailed knowledge maps. By providing a visual representation of citation networks, Cite Space facilitates an understanding of the evolving landscape of scientific research, highlighting key trends, pivotal studies, and emerging fields within the vast expanse of scholarly communication^44^.

Biblioshiny is a user-friendly web application featuring a graphical interface for the Bibliometrix R software, designed to simplify bibliometric analysis. It enables comprehensive evaluations and performs various functions, such as network analysis, descriptive data analysis, and visualization of bibliometric networks^45^.

## Results

### Search strategy and study selection

**Figure 1:**
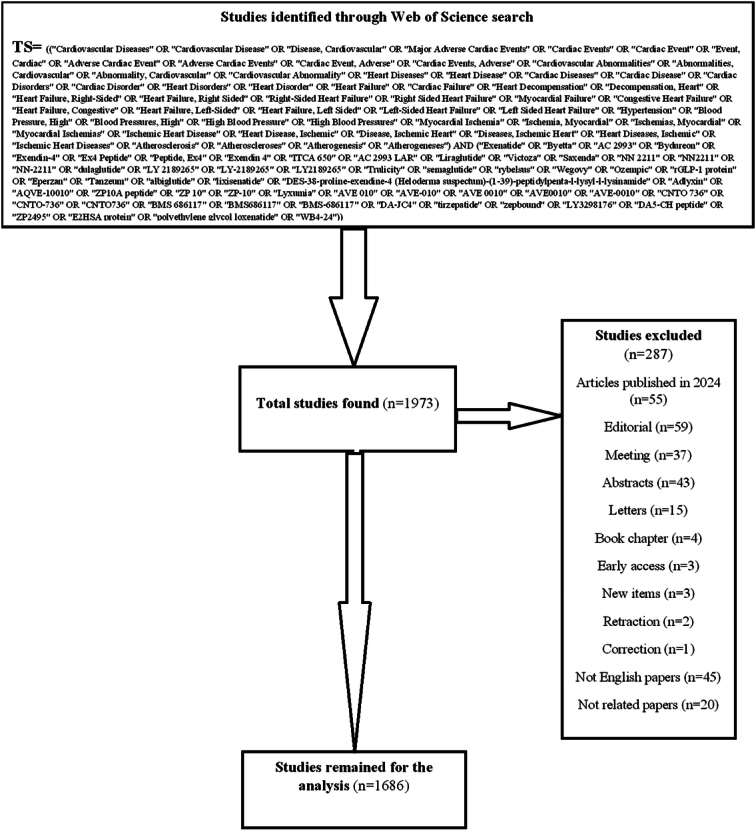
Study selection.

The Web of Science database was searched on 19 February 2024, and a total of 1973 articles were identified. After removing irrelevant articles, a total of 1686 articles were selected for this bibliometric analysis. The keywords used in the search included ‘Major Adverse Cardiac Events’, ‘Cardiac Events, Adverse’, ‘Heart Diseases’, ‘Heart Failure’, ‘Cardiac Failure’, ‘Hypertension’, ‘Ischemic Heart Disease’, ‘Exenatide’, ‘Liraglutide’, ‘Dulaglutide’, ‘Semaglutide’, ‘Albiglutide’, and ‘Tirzepatide’. The study selection process and the detailed search strategy are presented in Figure 1.

### Publication trends

**Figure 2:**
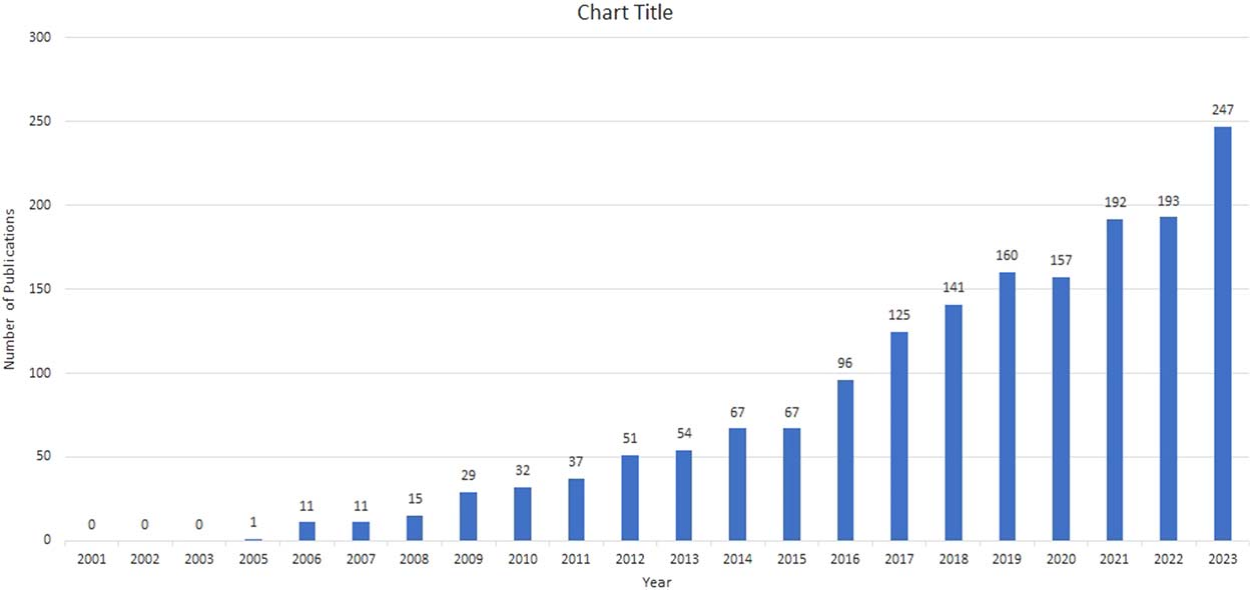
Trends of papers published regarding GLP-1 agonists and cardiovascular studies over the past 23 years.

The volume of publications within a specific timeframe is indicative of the evolving dynamics of research within this domain. As illustrated in Figure 2, from 2005 to 2023, there was a noticeable trend in the increase of studies dedicated to GLP-1 agonists within the field of cardiovascular research. The period from 2005 to 2015 witnessed a modest output of articles, suggesting that the exploration of GLP-1 agonists in cardiovascular science was at its early stages. However, between 2016 and 2019, there was a significant surge in scholarly contributions. Although there was a slight decline in 2020, the period from 2021 to 2023 experienced a marked and sustained increase in scholarly articles, peaking at 247 publications in 2023 alone. This trend highlights the growing interest and acknowledgment among researchers of the significant potential of GLP-1 agonists in cardiovascular health.

### Distribution of countries, regions, and institutions

**Figure 3:**
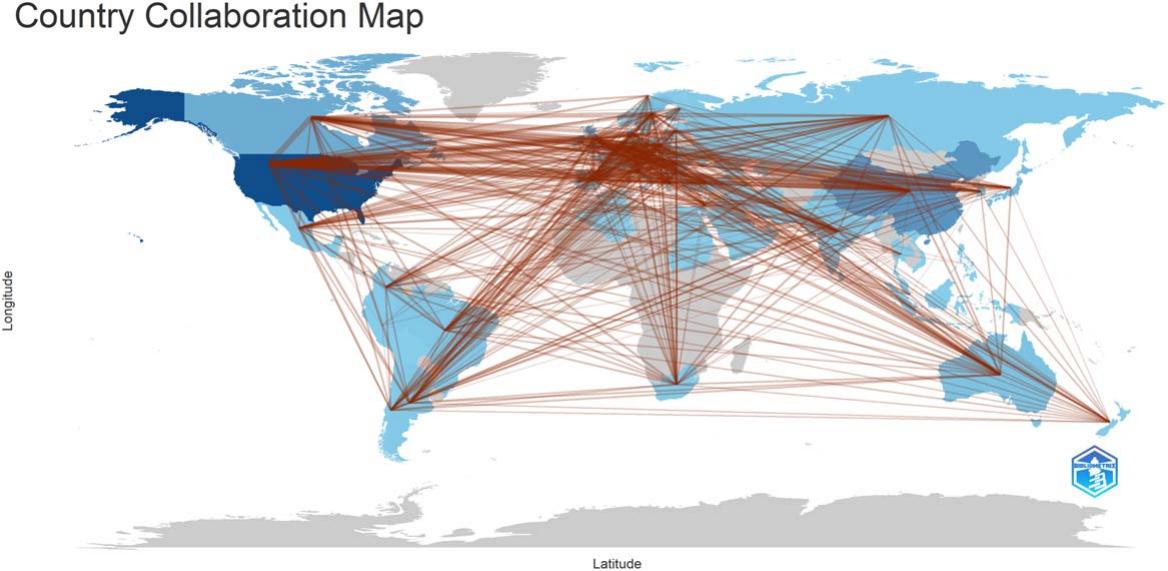
Countries collaboration in the world.

**Figure 4:**
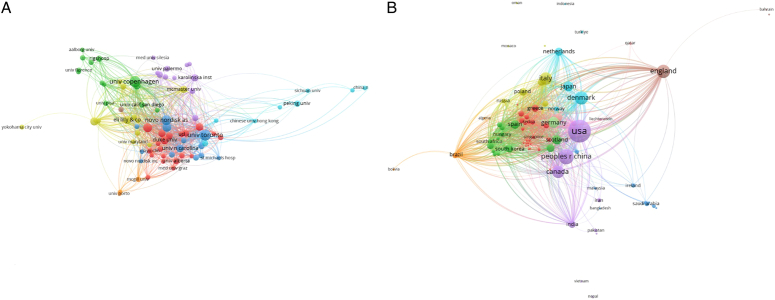
A: Geographical spread of publications across various institutions B: Geographical spread of publications across various nations.

**Figure 5:**
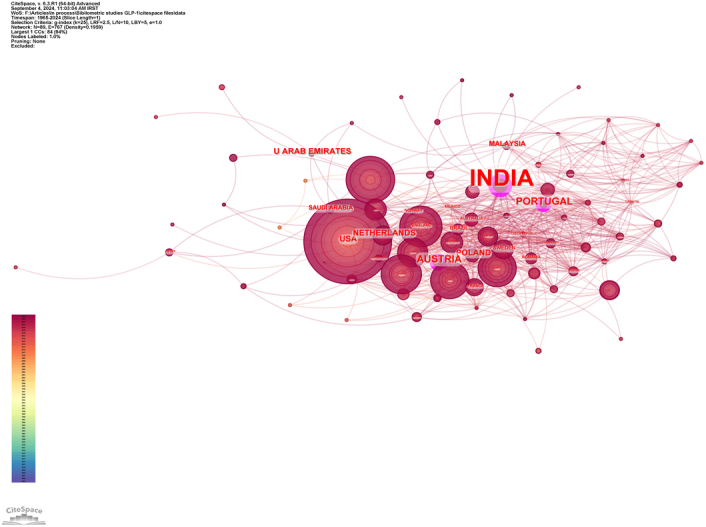
Counties with high centrality in the world.

A total of 1686 articles from 89 countries were published on the topic. Figure 3 shows the country's collaborations globally. The United States led with the highest number of publications, contributing 694 articles (41.16%), followed by China with 257 articles (15.24%), England with 199 articles (11.80%), Denmark with 191 articles (11.32%), and Canada with 167 articles (9.90%). The combined contributions from the United States and China accounted for more than half of the total publications. Among institutions, the University of Copenhagen and the University of Toronto were tied for the highest number of publications, each with 83 articles (4.92%), followed by Novo Nordisk with 62 articles (3.67%), Harvard Medical School with 51 articles (3.02%), and the University of Glasgow with 47 articles (2.78%). Table 1 presents the top 10 countries and institutions ranked by their contribution to the field in terms of the number of publications. Figures 4A and B imply that research on GLP-1 agonists conducted by the nations and organizations could have been pivotal in the field of cardiovascular studies. In this context, each node symbolizes a country/institution, with the node’s size reflecting the volume of published articles. The connections between nodes denote collaborations, with thicker connections indicating stronger collaborative ties. There exists a strong linkage between the USA and both Canada and England, as well as between China and the USA, and also between England and Denmark. Centrality demonstrates the impact of a country within the field. The analysis revealed that India exhibited the highest centrality score at 0.26, followed by Austria at 0.12, Portugal at 0.11, and the USA at 0.10 (Fig. 5).

**Table 1:** Leading countries and institutions in the field of GLP-1 agonists in cardiovascular disease.

Number	Country	Number of publications	Number of links	Institution	Number of publications	Number of links
1	United States	694	72	University of Copenhagen	83	47
2	China	257	60	University of Toronto	83	55
3	England	199	66	Novo Nordisk	62	37
4	Denmark	191	65	Harvard Medical School	51	51
5	Canada	167	64	The University of Glasgow	47	51
6	Italy	159	67	Eli Lilly and Company	41	28
7	Germany	123	63	Duke University	39	48
8	Japan	99	62	University of Washington	38	40
9	Scotland	76	61	University of North Carolina	36	39
10	Sweden	72	63	University of Oxford	32	33

### Journals and co-cited academic journals

The analysis unveiled that 520 scientific journals have disseminated research on GLP-1 agonists in the context of cardiovascular diseases. Notably, ‘Diabetes Obesity and Metabolism’ led in publications with 116 articles, accounting for 6.88% of the total, followed by ‘Cardiovascular Diabetology’ with 74 articles (4.38%), ‘Diabetes Therapy’ with 36 articles (2.13%), ‘Diabetes Care’ with 25 articles (1.48%), and ‘Diabetes Research and Clinical Practice’ also with 25 articles (1.48%). Table 2 presents the top 10 journals ranked by their contribution to the field in terms of the number of publications.

**Table 2:** Leading journals and co-cited journals in the field of GLP-1 agonists in cardiovascular disease.

Number	Journal name	Number of publications	Impact factor	JCR	Co-citation journal	Citation	Impact factor	JCR
1	Diabetes, Obesity and Metabolism	116	5.8	Q1	The New England Journal of Medicine	7579	158.5	Q1
2	Cardiovascular Diabetology	74	9.3	Q1	Diabetes Care	6709	16.2	Q1
3	Diabetes Therapy	36	3.8	Q2	The Lancet	4230	168.9	Q1
4	Diabetes Care	25	16.2	Q1	Diabetes, Obesity and Metabolism	3699	5.8	Q1
5	Diabetes Research and Clinical Practice	25	5.1	Q2	Circulation	2959	37.8	Q1
6	Nutrition, Metabolism & Cardiovascular Diseases	21	3.9	Q2	Diabetes	2566	7.7	Q1
7	Postgraduate Medicine	18	4.2	Q2	Diabetologia	2175	8.2	Q1
8	Diabetologia	17	8.2	Q1	Jama	1900	120.7	Q1
9	Biochemical and Biophysical Research Communications	16	3.1	Q2	The Lancet Diabetes and Endocrinology	1805	44.5	Q1
10	The Lancet Diabetes and Endocrinology	16	44.5	Q1	Cardiovascular Diabetology	1579	9.3	Q1

**Figure 6:**
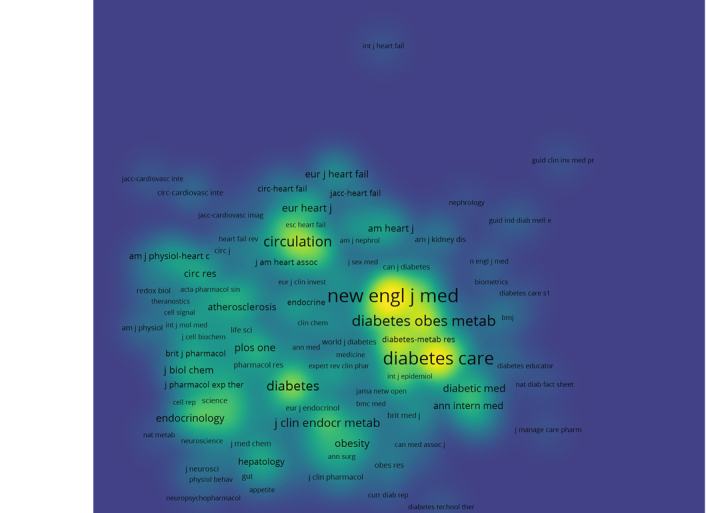
The density plot of the leading journals in the field of GLP-1 agonists in cardiovascular disease.

**Figure 7:**
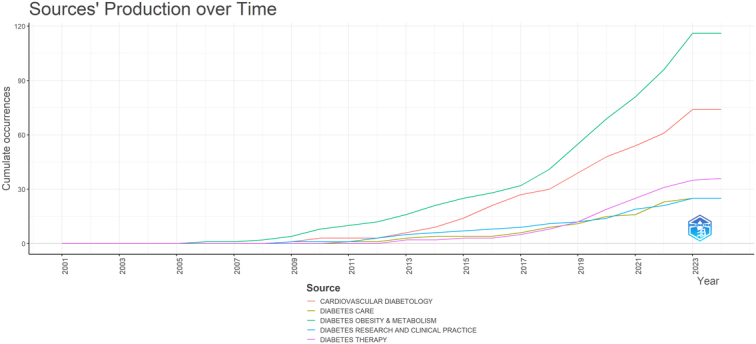
Journals’ production over time in the field of GLP-1 agonists in cardiovascular disease.

Cocitation analysis serves as a tool to uncover the interconnectedness among scholarly articles, illustrating how a journal’s influence within a specific research domain can be determined by its frequency of cocitation. This approach highlights the relational dynamics between publications, providing insights into the scholarly impact and thematic relevance of journals in the field. From our cocitation assessment, we identified 6898 journals that were cocited, with 10 of these journals being cited over 1500 times each. The ‘New England Journal of Medicine’ emerged as the most cited journal with 7579 citations, followed by ‘Diabetes Care’ with 6709 citations, ‘Lancet’ with 4230 citations, ‘Diabetes, Obesity and Metabolism’ with 3699 citations, and ‘Circulation’ with 2959 citations. Table 2 presents the top 10 leading co citied journals ranked by their contribution to the field. Figure 6 represents the density visualization plot of the co-cited journal. Figure 7 displays the journals’ production over time.

**Figure 8:**
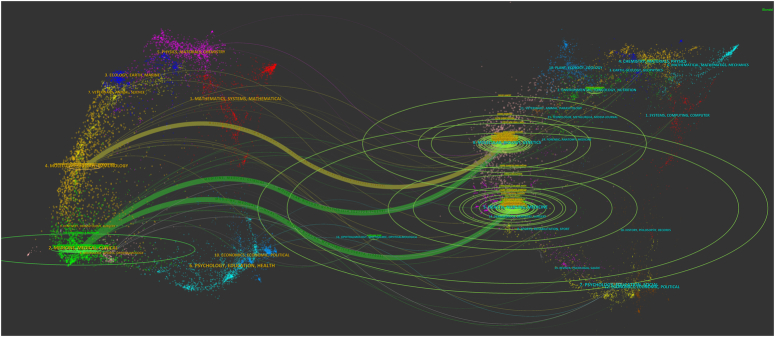
The dual-map overlay of journals on GLP-1 agonists in cardiovascular diseases.

The dual-map overlay representing journal relationships showcases the citation dynamics, with citing journals positioned on the left and cited journals on the right. The visualization, highlighted in Figure 8, revealed three primary citation trajectories, marked by two green paths and one yellow path. The dual-map overlay illustrated that research articles from Health/Nursing/Medicine journals received significant citations from publications in the Medicine/Medical/Clinical sector. Additionally, works published in Molecular/Biology/Genetics journals were frequently cited by both Molecular/Biology/Immunology and Medicine/Medical/Clinical journals. This mapping highlights the interconnectedness and impact of these fields on one another, underscoring the cross-disciplinary nature of scientific research and its dissemination.

This mapping highlights the interdisciplinary character of scientific research and its dissemination by highlighting the relationships between and effects of several domains on one another.

### Authors and cocited authors

A total of 10 005 authors have contributed to the domain of GLP-1 agonists and cardiovascular diseases. Notably, John B. Buse led with the highest number of published papers (*n*=30, 1.77%), followed by Lawrence A. Leiter (*n*=26, 1.54%), Adrian F. Hernandez (*n*=24, 1.42%), Hertzel Gerstein (*n*=22, 1.30%), and Michael A. Nauck (*n*=20, 1.18%).

In terms of citations, John B. Buse also maintained the lead with an impressive count of 12 839, followed by Bernard Zinman (*n*=7576), Steven P. Marso (*n*=7380), Michael A. Nauck (*n*=6353), and Neil Poulter (*n*=6063).

**Figure 9:**
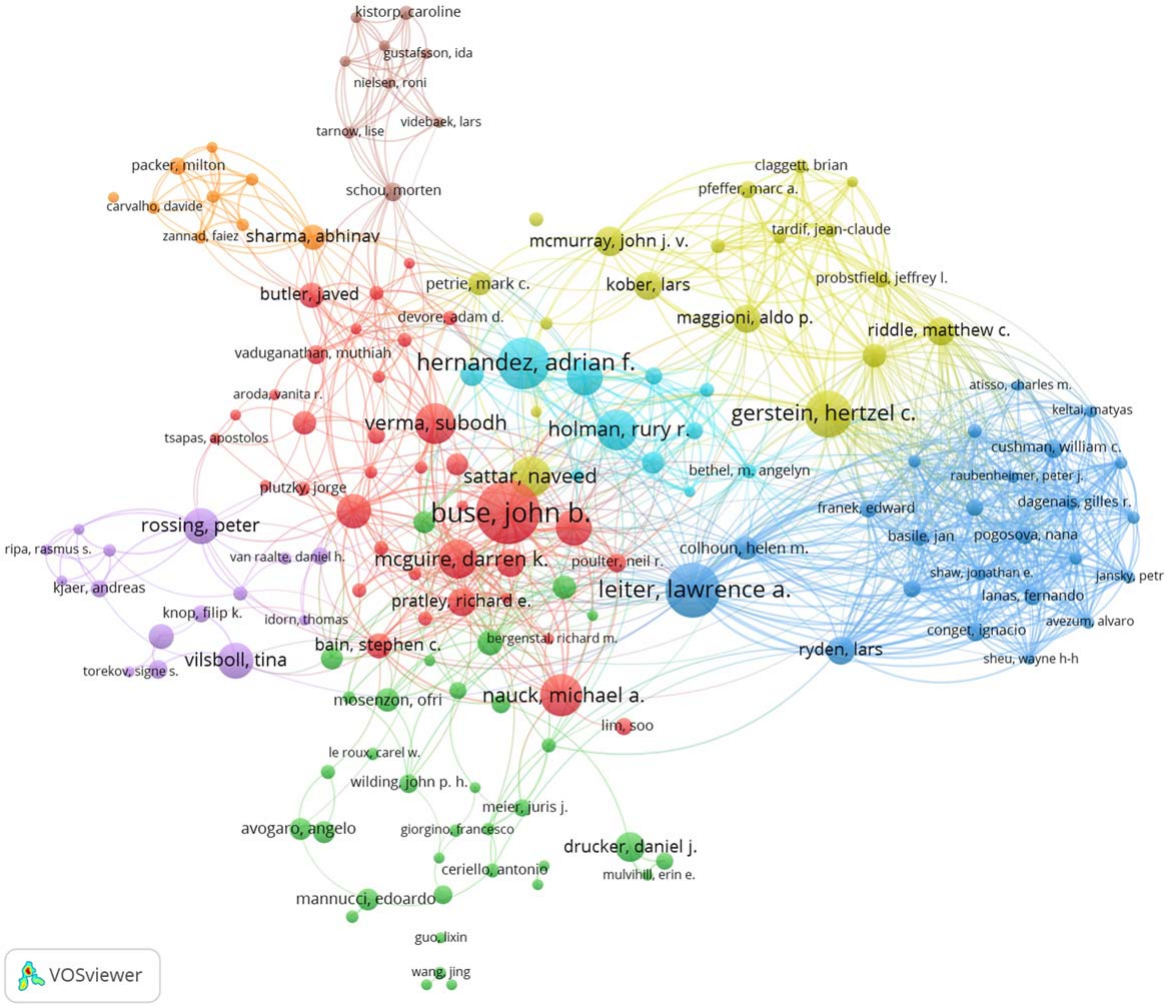
Co-authorship visualization on GLP-1 agonists in cardiovascular diseases.

Moreover, the results of co-authorship analysis indicated a robust collaboration among authors within this field. Hertzel Gerstein, Lawrence A. Leiter, Lars Ryden, Matthew Riddle, and John B. Buse emerged as key collaborators, significantly contributing to advancements in this domain. Figure 9 showcases the co-authorship network among contributing authors in this field.

When two or more authors are cited together, they are referred to as cocited authors. Among 30 525 co-cited authors, 94 individuals stood out with over 100 citations each. Steven P. Marso emerged as the most cocited author with a count of 1036 citations, followed by Hertzel Gerstein (*n*=807), Michael A. Nauck (*n*=628), Daniel Drucker (*n*=625), and Bernard Zinman (*n*=619). These authors’ frequent cocitations highlight their significant contributions and influence within the field. Table 3 represents the top 10 leading authors and cocited authors.

**Table 3:** Top 10 leading authors and co-cited authors.

Number	Author with high number of publications	Number of publications	Author with high number of citations	Number of citations	The most co-cited authors	Number of citations
1	John B. Buse	30	John B. Buse	12839	Steven P. Marso	1036
2	Lawrence A. Leiter	26	Bernard Zinman	7576	HC Gerstein	807
3	Adrian F. Hernandez	24	Steven P. Marso	7380	Michael A. Nauck	628
4	Hertzel Gerstein	22	Michael A. Nauck	6353	Daniel Drucker	625
5	Michael A. Nauck	20	Neil Poulter	6063	Bernard Zinman	619
6	Rury Holman	19	Peter Rossing	5208	Julio Rosenstock	611
7	Subodh Verma	19	Richard M. Bergenstal	5115	John B. Buse	589
8	Naveed Sattar	19	Johannes F E Mann	5093	Rury R Holman	581
9	Darren McGuire	18	Apostolos Tsapas	5077	Ralph A. DeFronzo	388
10	Robert John Mentz	17	Kirstine Brown-Frandsen	5032	Marc A Pfeffer	387

### Cited and cocited references

**Figure 10:**
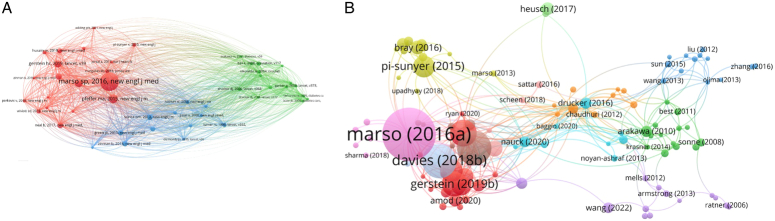
A: Co-cited references network visualization. B: Most cited articles network visualization.

**Figure 11:**
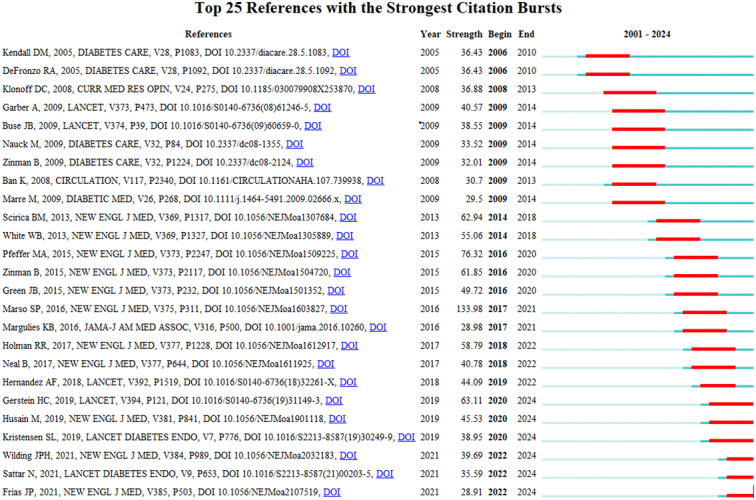
Top papers with citation burst time in the field of GLP-1 agonists in cardiovascular disease.

The cocitation analysis uncovers that when two references are cited together within another article’s bibliography, it forms a cocitation link between those references. From a total of 48 622 identified co-cited references, 46 were cited at least 100 times, as depicted in Figure 10A. This study further narrowed down to the top 10 cited references. Out of 1686 included studies, 116 were cited at least 100 times by other scholars, as shown in Figure 10B. Table 4 provides a detailed list of the top 10 cited and cocited references in this area, offering insights into the most influential works and their interconnections. Figure 11 shows the top 25 papers with citation burst.

**Table 4:** Top 10 cited and co-cited references.

Number	Title of most cited paper	Doi	Published year	Title of most co-cited paper	Doi	Published year
1	Liraglutide and Cardiovascular Outcomes in Type 2 Diabetes	10.1056/NEJMoa1603827	2016	Liraglutide and Cardiovascular Outcomes in Type 2 Diabetes	10.1056/NEJMoa1603827	2016
2	Management of Hyperglycemia in Type 2 Diabetes, 2018. A Consensus Report by the American Diabetes Association (ADA) and the European Association for the Study of Diabetes (EASD)	10.2337/dci18-0033	2018	Lixisenatide in Patients with Type 2 Diabetes and Acute Coronary Syndrome	10.1056/nejmoa1509225	2015
3	Lixisenatide in Patients with Type 2 Diabetes and Acute Coronary Syndrome	10.1056/NEJMoa1509225	2015	Effects of Once-Weekly Exenatide on Cardiovascular Outcomes in Type 2 Diabetes	10.1056/nejmoa1612917	2017
4	Dulaglutide and cardiovascular outcomes in type 2 diabetes (REWIND): a double-blind, randomised placebo-controlled trial	10.1016/S0140-6736(19)31149-3	2019	Canagliflozin and Cardiovascular and Renal Events in Type 2 Diabetes	10.1056/nejmoa1611925	2017
5	Effects of Once-Weekly Exenatide on Cardiovascular Outcomes in Type 2 Diabetes	10.1056/NEJMoa1612917	2017	Dulaglutide and cardiovascular outcomes in type 2 diabetes (REWIND): a double-blind, randomised placebo-controlled trial	10.1016/s0140-6736(19)31149-3	2019
6	A Randomized, Controlled Trial of 3.0 mg of Liraglutide in Weight Management	10.1056/NEJMoa1411892	2015	Albiglutide and cardiovascular outcomes in patients with type 2 diabetes and cardiovascular disease (Harmony Outcomes): a double-blind, randomised placebo-controlled trial	10.1016/s0140-6736(18)32261-x	2018
7	Albiglutide and cardiovascular outcomes in patients with type 2 diabetes and cardiovascular disease (Harmony Outcomes): a double-blind, randomised placebo-controlled trial	10.1016/s0140-6736(18)32261-x	2018	Saxagliptin and Cardiovascular Outcomes in Patients with Type 2 Diabetes Mellitus	10.1056/nejmoa1307684	2013
8	Cardiovascular, mortality, and kidney outcomes with GLP-1 receptor agonists in patients with type 2 diabetes: a systematic review and meta-analysis of cardiovascular outcome trials	10.1016/S2213-8587(19)30249-9	2019	Effect of Sitagliptin on Cardiovascular Outcomes in Type 2 Diabetes	10.1056/nejmoa1501352	2015
9	Semaglutide and Cardiovascular Outcomes in Patients with Type 2 Diabetes	10.1056/NEJMoa1607141	2016	Alogliptin after Acute Coronary Syndrome in Patients with Type 2 Diabetes	10.1056/nejmoa1305889	2013
10	Management of obesity	10.1016/S0140-6736(16)00271-3	2016	Empagliflozin, Cardiovascular Outcomes, and Mortality in Type 2 Diabetes	10.1056/nejmoa1504720	2015

### Keyword trends

**Figure 12:**
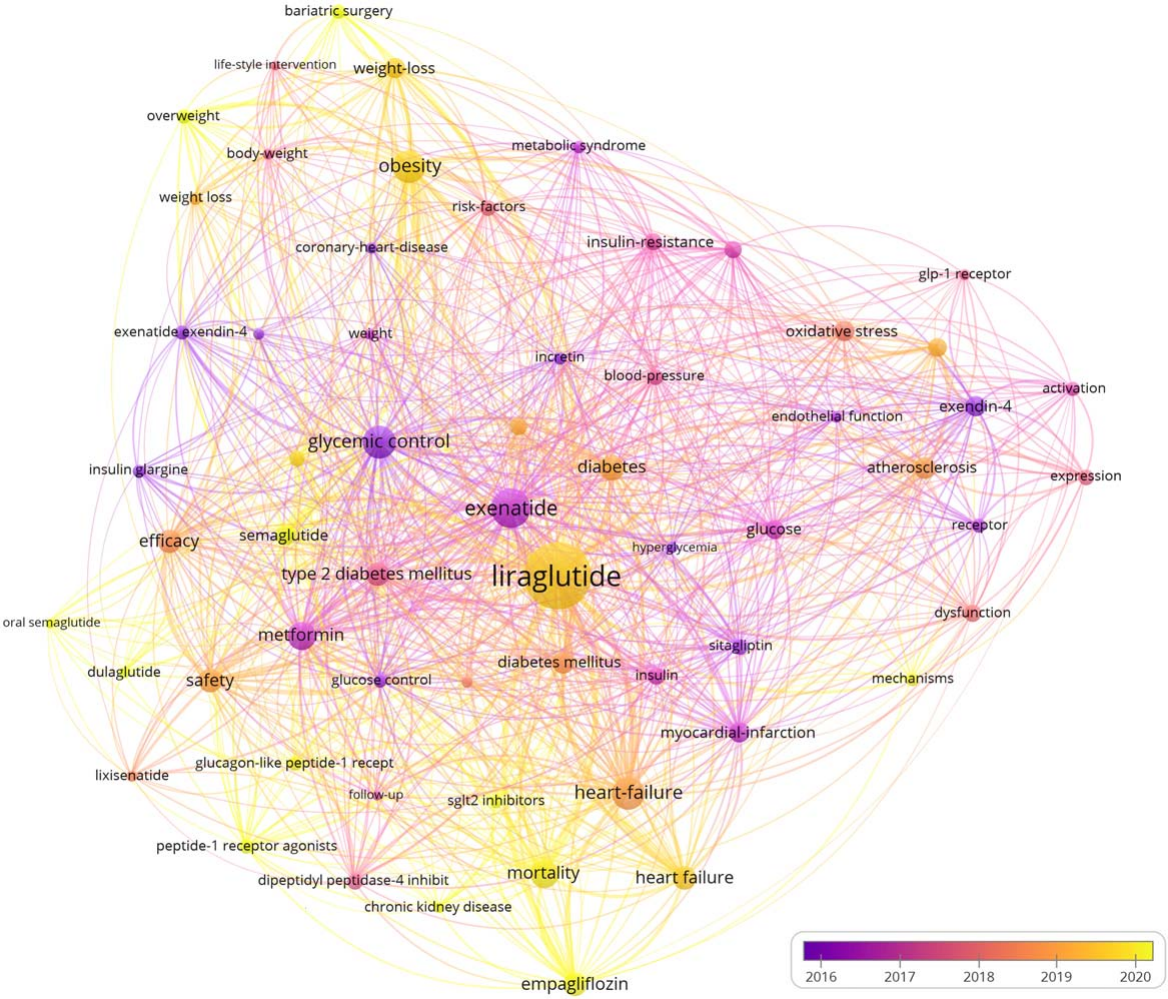
Overlay visualization of the co-occurrence keywords.

**Figure 13:**
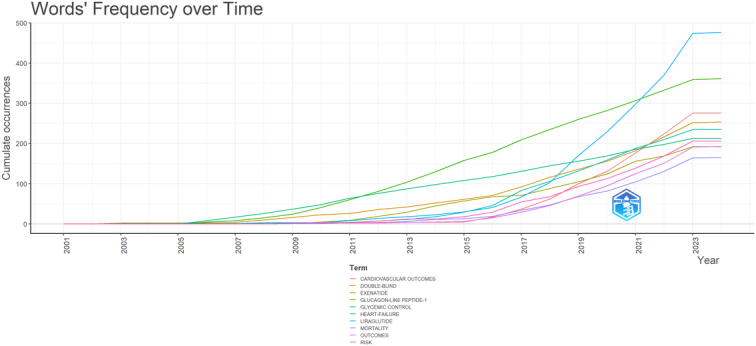
Keywords’ frequency overtime in the field of GLP-1 agonists in cardiovascular diseases.

In our analysis, we identified a total of 4624 keywords across the included studies. Through cooccurrence keyword analysis, we found that ‘liraglutide’ (*n*=708), ‘exenatide’ (*n*=301), ‘obesity’ (*n*=235), ‘heart failure’ (*n*=235), and ‘glycemic control’ (*n*=224) emerged as the most frequently repeated keywords. Moreover, the overlay visualization plot revealed that keywords such as ‘tirzepatide’, ‘semaglutide 2.4 mg’, ‘HFpEF’, ‘kidney outcomes’, and ‘finerenone’ are recent keywords with a higher average publication year, indicating their prominence in the field of GLP-1 agonists in cardiovascular disease (Fig. 12). Figure 13 showed the keywords’ frequency overtime.

### Cluster analysis

**Figure 14:**
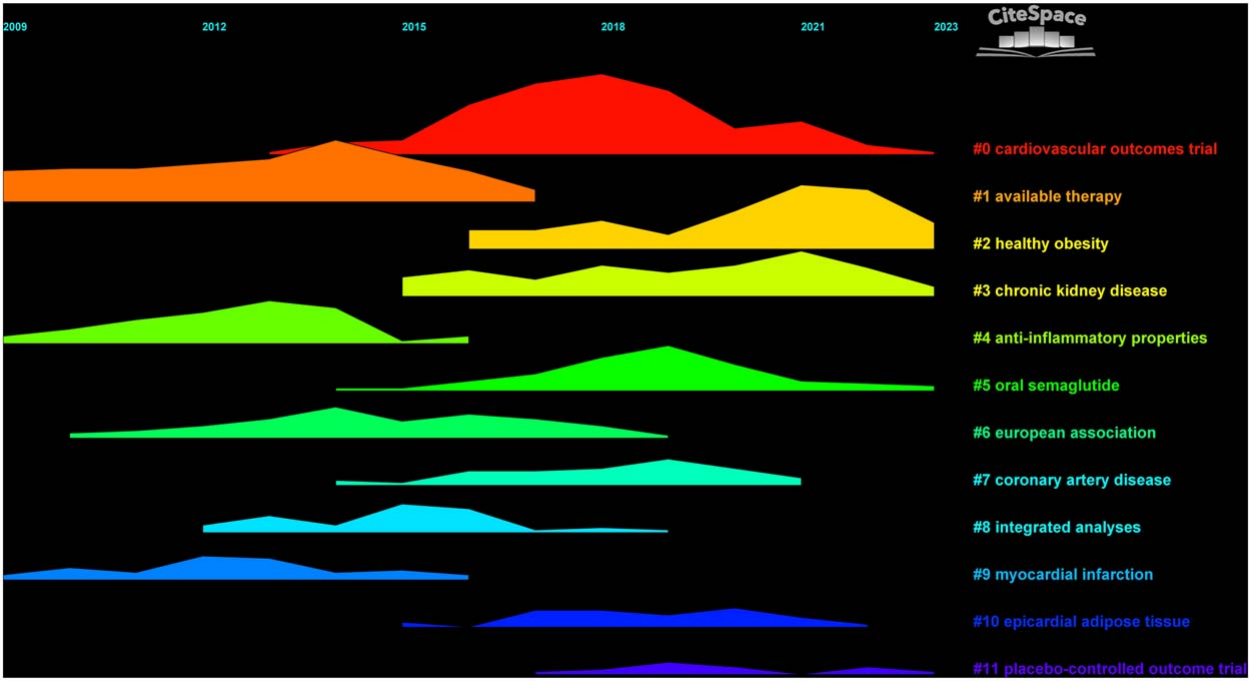
Time line cluster analysis.

The cluster analysis conducted within the research domain of GLP-1 agonists and their impact on cardiovascular diseases has led to a significant classification, identifying 12 distinct clusters. Initially, the focus was primarily on the available therapeutic options and their anti-inflammatory properties. However, recent trends have shifted attention towards emerging hotspots within this field. Notably, topics such as ‘healthy obesity’, ‘oral semaglutide’, ‘cardiovascular outcomes’, and ‘chronic kidney disease’ have gained prominence, marking them as current areas of intense research interest (Fig. 14).

## Discussion

Continued high levels of blood sugar cause harm to the microvasculature, resulting in complications like retinopathy, neuropathy, and nephropathy in patients with T2DM. Notably, chronic hyperglycemia is also identified as a contributor to cardiovascular (CV) risk. Moreover, T2DM frequently coincides with obesity, dyslipidemia, hypertension, and inflammation, all of which are standalone CV risk factors. Consequently, T2DM substantially elevates the risk of CVDs by two to five times^46–50^.

Interestingly, following conflicting outcomes in extensive trials examining the impact of glucose reduction on CVD risks, current consensus suggests that lowering glucose might not be significantly crucial for preventing CVD in T2DM. Contemporary guidelines advise healthcare providers to lower glucose levels for microvascular disease prevention but emphasize a personalized evaluation of CVD risk factors. Notably, managing blood pressure, lipids, and, if necessary, antiplatelet therapy are emphasized. Despite these interventions, substantial CVD risk persists, underscoring the imperative for novel treatment approaches to effectively address and prevent CVD in individuals with T2DM^51–53^.

The identification of GLP-1 receptors (GLP-1Rs) on cardiomyocytes has generated significant interest among clinicians and researchers regarding potential cardiovascular advantages associated with GLP-1 and GLP-1-based therapies. Initial clinical trials have indicated potential positive impacts of GLP-1 and its therapies on factors such as cholesterol levels, blood pressure, microcirculation, and low-grade inflammation^54–65^. Notably, larger randomized CV outcome trials have provided substantial evidence that GLP-1 receptor agonists effectively decrease CVDs in patients with T2DM^29,64,66–70^.

The bibliometric analysis presented in this study highlighted an increasing trajectory of research interest in GLP-1 agonists, particularly within the realm of cardiovascular health, over the past two decades. This surge in scholarly publications, especially notable from 2016 onwards, reflects a broader recognition within the scientific community of the potential benefits these agents may offer in cardiovascular disease management and prevention.

Innovative clinical trials highlighting the cardiovascular benefits of GLP-1 agonists were reported in 2016. The SUSTAIN-6 experiment was a randomized controlled trial (RCT) that included 3297 individuals with type 2 diabetes mellitus and was published in the New England Journal of Medicine in 2016. For 104 weeks, participants were randomly assigned to receive either a placebo or 0.5 mg or 1.0 mg of semaglutide once a week. This experiment showed that semaglutide recipients had a significantly lower rate of cardiovascular death, nonfatal myocardial infarction, or nonfatal stroke than the placebo group in individuals with type 2 diabetes who were at high cardiovascular risk^66^. The LEADER study, another noteworthy trial, was also published in the New England Journal of Medicine in 2016. Nine thousand three hundred forty patients with type 2 diabetes mellitus participated in this study, and the results indicated that patients receiving liraglutide had a lower rate of the first occurrence of death from cardiovascular causes, nonfatal myocardial infarction, or nonfatal stroke than patients receiving placebo^67^. On the other hand, the NHLBI trial, which was reported in JAMA in 2016, assessed 300 hospitalized patients who had low left ventricular ejection fraction (LVEF) and heart failure. According to this study, these patients’ posthospitalization clinical stability did not improve when liraglutide was used^71^. Together, these studies demonstrated the potential cardiovascular advantages of GLP-1 agonists, especially liraglutide and semaglutide, for individuals with type 2 diabetes. The results caused further investigation and debate on the usage of these drugs to lower cardiovascular risks.

Several factors may contribute to heightened interest in GLP-1 agonists. First, the pathophysiological understanding of CVDs has evolved significantly, with increasing recognition of the role of inflammation, metabolic dysregulation, and insulin resistance in the progression of these conditions^72–74^. GLP-1 agonists, initially developed for the treatment of type 2 diabetes, have shown promise in addressing these underlying mechanisms, offering a novel therapeutic angle for CVD management^75^.

Moreover, pivotal clinical trials conducted during this period have played a crucial role in shaping the research landscape. Studies such as the LEADER and SUSTAIN-6 have demonstrated the efficacy of GLP-1 agonists in reducing adverse cardiovascular events in individuals with type 2 diabetes^66,67^. These findings have spurred further investigation into the mechanisms of action, optimal use, and potential benefits of GLP-1 agonists beyond glucose regulation.

The slight decline in publications observed in 2020 might be attributed to the global shift in research priorities due to the COVID-19 pandemic, which impacted various fields of scientific inquiry. Nonetheless, the rebound in scholarly output from 2021 to 2023 underscores the resilience and sustained interest of the cardiovascular research community in exploring GLP-1 agonists. The peak in publications in 2023 could also reflect the culmination of long-term research projects initiated in response to earlier clinical findings, alongside the increasing availability of GLP-1 agonists as therapeutic options.

Our investigation indicated a significant concentration of scholarly publications emanating from Europe and North America, highlighting an imbalance in the distribution of academic resources predominantly in favor of developed nations. This trend is mirrored in the formation of numerous research collectives within these regions. Among these, groups led by Professor John B. Buse stand out as prominent clusters in their field. Professor Buse has played a pivotal role in numerous guideline and international consensus panels, steering several extensive multicenter, prospective, randomized controlled trials, and systematic reviews focusing on GLP1RA’s impact on cardiovascular outcomes^76–78^.

Dr. Lawrence A. Leiter holds several prestigious positions, including the directorship of the Lipid Clinic, the role of associate director at the Clinical Nutrition and Risk Factor Modification Centre, and associate scientist at the Li Ka Shing Knowledge Institute, all at St. Michael’s Hospital in Toronto. Previously, he led the Division of Endocrinology and Metabolism at the same institution. As a professor affiliated with the Departments of Medicine and Nutritional Sciences at the University of Toronto, Dr Leiter is deeply involved in research. His work focuses on clinical trials aimed at the prevention of atherosclerosis, particularly in relation to diabetes, and extends to exploring dietary and pharmacological strategies for managing diabetes mellitus, hyperlipidemia, hypertension, and obesity^66,79–81^.

Adrian F. Hernandez, a cardiologist within the Division of Cardiology at Duke University Medical Center, specialized in heart failure research. He has also made significant contributions to various randomized controlled trials, playing a pivotal role in this critical research area^64,71^.

Based on bibliometric analysis, it was found that there was a significant level of collaboration among multiple institutions located in different countries. The shift towards collaborative and interdisciplinary research has played a pivotal role in evolving this dynamic field. Collaboration among authors and institutions has facilitated a rich exchange of ideas, resources, and expertise. Such partnerships have accelerated the pace of discovery, enabling the execution of large-scale clinical trials and comprehensive studies that span across disciplines. The integration of knowledge from endocrinology, cardiology, pharmacology, and molecular biology, fostered by these collaborative efforts, has been instrumental in advancing our understanding of how GLP-1 agonists can be beneficial for cardiovascular health.

Our examination of cocited references revealed that the LEADER trial stood in the top position, followed by the ELIXA trial in second place and the EXCEL trial in third. The REWIND trial came in fifth, while the Harmony trial was ranked sixth. These are all critical studies investigating the impact of GLP-1 agonists on cardiovascular outcomes. Authored by esteemed experts across various cardiology and endocrinology research centers, these seminal papers have been published in prestigious journals^64,67,68,82,83^.

Additionally, the fourth most cocited reference is the CANVAS study, which evaluated the effects of canagliflozin, a sodium-glucose co-transporter 2 (SGLT2) inhibitor. The seventh most cited reference was the SAVOR-TIMI trial, examining the impact of Saxagliptin, a dipeptidyl peptidase-4 (DPP-4) inhibitor, on diabetic patients^84,85^. These findings underscore the expanding literature on the outcomes associated with SGLT2 and DPP-4 inhibitors, in conjunction with GLP-1 agonists.

The bibliometric analysis of keyword trends highlighted ‘tirzepatide’ and ‘semaglutide 2.4 mg’ as the most current keywords, with ‘healthy obesity’ and ‘chronic kidney disease (CKD)’ emerging as the latest clusters. A landmark study by Garvey *et al*., from the SURMOUNT-2 trial, marks the first investigation of tirzepatide in adults with obesity and type 2 diabetes. This 72-week trial study demonstrated that tirzepatide, administered once weekly at doses of 10 mg and 15 mg, significantly reduced body weight while maintaining a safety profile consistent with other incretin-based weight management therapies^86^. In parallel, the STEP trials investigating semaglutide have yielded noteworthy results; particularly, the recently published STEP 5 trial revealed that in adults who are overweight (with at least one weight-related comorbidity) or obese, semaglutide treatment facilitated significant weight loss over a period of 104 weeks when compared to placebo^87^.

The idea of metabolically healthy obesity (MHO) suggests that there exists a subset of obese people who do not exhibit the typical cardio-metabolic risk factors often found in obese individuals with dysfunctional adipose tissue and insulin resistance, a condition referred to as metabolic syndrome or the metabolically unhealthy obesity (MUO) phenotype. Those with MHO seem to have better functioning adipose tissue and exhibit greater insulin sensitivity^88^.

Recent studies have highlighted the promising role of the new GLP-1 agonist drug, Tirzepatide, in managing MHO. Recognized increasingly as a critical risk factor, MHO is linked to the development of cardiovascular diseases, cerebrovascular conditions, and peripheral artery disease. Furthermore, individuals with MHO are at heightened risk of transitioning to a metabolically unhealthy phenotype. Tirzepatide has demonstrated remarkable efficacy, showcasing significant benefits such as weight reduction and notable improvements in key metabolic markers including hemoglobin A1c, fasting serum glucose, and triglyceride/lipoprotein levels. These outcomes suggest that Tirzepatide holds considerable potential in aiding individuals who are either metabolically healthy obese or overweight, reducing their risk of adverse cardiovascular events and preventing the shift towards a metabolically unhealthy status^89^.

The presence of CKD in the context of GLP-1 agonist research is noteworthy, given the established cardiovascular benefits of these agents in individuals with type 2 diabetes, a common precursor to both CKD and cardiovascular diseases^90–92^. This cluster’s emergence points to an expanding interest in the renal outcomes associated with GLP-1 agonist therapy, reflecting a broader understanding of the cardiorenal axis in metabolic diseases. The exploration of GLP-1 agonists in CKD patients may offer valuable insights into their potential to slow the progression of renal decline and mitigate cardiovascular risk^93–96^. This is particularly relevant given the increased cardiovascular morbidity and mortality associated with CKD^97^. GLP-1 agonists have emerged as promising therapeutic agents in the management of patients with CKD, particularly those with advanced stages. While their impact on cardiovascular outcomes in CKD patients with an estimated glomerular filtration rate (eGFR) below 30 has been less pronounced, they exhibit notable renal protective effects. Studies such as the LEADER, ELIXA, REWIND, and SUSTAIN-6 trials have consistently demonstrated benefits in renal outcomes among patients with relatively fair kidney function, with a significant reduction in parameters such as new-onset macroalbuminuria and decline in eGFR^66,67,98,99^. These effects are attributed to various mechanisms, including the reduction of oxidative stress-induced autophagy, endothelial dysfunction, and local inflammation. Additionally, GLP-1 agonists have been shown to enhance natriuresis and potentially protect the kidneys via the sodium-hydrogen exchanger 3 (NHE3) pathway^100–102^. Moreover, despite previous trials excluding patients with advanced CKD, recent studies have started to shed light on the renal protective effects of GLP-1 agonists even in this population. The findings suggest a delay in the progression to end-stage renal disease (ESRD) and a slower decline in kidney function, as evidenced by a significant difference in the time to initiation of dialysis between GLP-1 agonist-treated patients and those receiving other antidiabetic agents^66,67^. However, the exact mechanisms underlying these renal protective effects in advanced CKD remain unclear and warrant further investigation. Nevertheless, these findings hold promise for improving the management and prognosis of CKD patients, especially those with type 2 diabetes, by offering a potential therapeutic avenue that not only addresses glycemic control but also mitigates renal complications.

Similar to our study, other bibliometric research has also assessed GLP-1 agonists. Dagli *et al*. conducted a similar bibliometric analysis focused specifically on Semaglutide as a GLP-1 agonist. Their study examined publication trends from 2014 to 2022, revealing a steady rise in research output, peaking in 2022. They identified key authors, institutions, and research themes related to Semaglutide efficacy in glycemic control and broader diabetes management implications^103^.

In contrast, our analysis examined a broader landscape of GLP-1 agonists and their cardiovascular applications, spanning a longer timeframe from 2005 to 2023. We observed a more gradual increase in publications from 2005 to 2015, followed by a significant surge from 2016 to 2019. This suggests the field has evolved, with growing recognition of the cardiovascular benefits of GLP-1 agonists beyond just their antidiabetic effects.

Additionally, Shou *et al*., analyzed GLP-1 agonist publications from 2005 to 2021, reporting a similar trajectory to our findings. They noted a gradual increase until 2012, a slight dip, and then a substantial surge from 2016 to 2020, peaking in 2020. This corroborates the trends we observed in our analysis, indicating a sustained and intensifying research interest in this area over the past decade^104^.

The differences in our findings compared to previous studies can be attributed to the broader scope of our analysis, which encompasses the entire GLP-1 agonist class and its cardiovascular applications, rather than focusing on a single agent. This allowed us to capture the dynamic evolution of the research landscape and the changing emphasis on the cardioprotective properties of these therapies.

This bibliometric study provided a comprehensive examination of the research landscape surrounding GLP-1 agonists and their impact on cardiovascular diseases. The search strategy, which spanned multiple decades without any time restriction, ensured that the analysis captured the full breadth and evolution of this field. The utilization of well-established bibliometric tools, such as VOS viewer and Cite Space, further strengthened the reliability of findings. The multifaceted approach, incorporating analyses of publication trends, geographical distributions, influential journals, key authors, landmark citations, and thematic developments, offered a multidimensional perspective on the state and future directions of this research domain. The integration of both quantitative and visual techniques allowed for the clear identification of research hotspots, collaborative networks, and emerging areas of interest. However, the study was not without its limitations. The reliance on the Web of Science Core Collection as the sole data source may have limited the inclusion of relevant publications indexed in other scholarly databases. Additionally, the search strategy, while comprehensive, may have excluded some studies that did not explicitly mention ‘GLP-1 agonists’ or ‘cardiovascular diseases’ in the title, abstract, or keywords. Integrating qualitative methods, such as manual review of a sample of publications or expert interviews, could have provided additional contextual understanding of the research dynamics and emerging themes.

## Conclusions

GLP-1 agonists have emerged as a cornerstone in the therapeutic management of patients with T2DM, gaining prominence for their cardioprotective properties. These benefits have sparked a surge in research and publications focused on the cardioprotective effects of GLP-1 agonists, witnessing a significant increase over the past two decades. Numerous trials investigating these effects have been conducted, yielding results that have been featured in prestigious journals. This research has been characterized by a collaborative effort among authors from various institutions and countries, highlighting the global interest and effort in understanding and leveraging the benefits of GLP-1 agonists for patients with T2DM.

Recent studies have concentrated on innovative GLP-1 agonists such as tirzepatide and semaglutide 2.4 mg, which represent the latest advancements in this field. The exploration of these medications extends to new frontiers in diabetes management, including their impact on individuals with ‘healthy obesity’ and CKD, areas that have attracted considerable research attention. These developments underscore the evolving landscape of T2DM treatment, where the focus is not only on glucose control but also on addressing the broader cardiovascular and renal complications associated with the disease. The continuous exploration of GLP-1 agonists and their multifaceted benefits marks a significant stride toward comprehensive management strategies for patients with type 2 diabetes mellitus.

## Ethical approval

Since we conducted a bibliometric study, we did not have any human or animal subjects and our study did not require an ethical approval.

## Consent

Since we did not have any human or animal subject, conducting this section is not applicable.

## Source of funding

There is no funding for the present study.

## Author contribution

A.M. and E.A.S.: provided the research idea; E.A.S. and S.H.: designed the study; E.A.S. and S.O.: performed the analysis; M.H.K. and S.J.: conducted the search strategy; A.M. and R.R.K.: designed the illustrations. All authors contributed to drafting the manuscript.

## Conflicts of interest disclosure

The authors declare no competing interest.

## Research registration unique identifying number (UIN)

The current study is a bibliometric study and UIN is not applicable for this study.

## Guarantor

Ehsan Amini-Salehi (MD), Guilan University of Medical Sciences, 41448-95655, Rasht, Iran. Tel.: +98 1315535116, fax: +98 1315534951. E-mail: ehsanaminisalehi1998@gmail.com

## Data availability statement

Data from the study can be provided by the corresponding author on reasonable request.

## Provenance and peer review

Not commissioned, externally peer-reviewed.

## Supplementary Material

## References

[1] Popoviciu MS Păduraru L Yahya G . Emerging role of GLP-1 agonists in obesity: a comprehensive review of randomised controlled trials. Int J Mol Sci 2023;24:10449. 37445623 10.3390/ijms241310449 PMC10341852

[2] Hinnen D . Glucagon-like peptide 1 receptor agonists for type 2 diabetes. Diabetes Spectr 2017;30:202–210. 28848315 10.2337/ds16-0026 PMC5556578

[3] Piccini S Favacchio G Panico C . Time-dependent effect of GLP-1 receptor agonists on cardiovascular benefits: a real-world study. Cardiovasc Diabetol 2023;22:69. 36966321 10.1186/s12933-023-01800-z PMC10039680

[4] Tan X Liang Y Rajpura JR . Once-weekly glucagon-like peptide-1 receptor agonists vs dipeptidyl peptidase-4 inhibitors: cardiovascular effects in people with diabetes and cardiovascular disease. Cardiovasc Diabetol 2023;22:319. 37985992 10.1186/s12933-023-02051-8 PMC10662529

[5] Drucker DJ . Mechanisms of action and therapeutic application of glucagon-like peptide-1. Cell Metab 2018;27:740–756. 29617641 10.1016/j.cmet.2018.03.001

[6] Shaefer CF Jr Kushner P Aguilar R . User’s guide to mechanism of action and clinical use of GLP-1 receptor agonists. Postgrad Med 2015;127:818–826. 26371721 10.1080/00325481.2015.1090295

[7] Nevola R Epifani R Imbriani S . GLP-1 receptor agonists in non-alcoholic fatty liver disease: current evidence and future perspectives. Int J Mol Sci 2023;24:1703. 36675217 10.3390/ijms24021703 PMC9865319

[8] Mantovani A Petracca G Beatrice G . Glucagon-like peptide-1 receptor agonists for treatment of nonalcoholic fatty liver disease and nonalcoholic steatohepatitis: an updated meta-analysis of randomized controlled trials. Metabolites 2021;11:73. 33513761 10.3390/metabo11020073 PMC7911747

[9] Amini-Salehi E Letafatkar N Norouzi N . Global prevalence of nonalcoholic fatty liver disease: an updated meta-analysis on 78 million population over 38 countries. Arch Med Res 2024;55:103043. 39094335 10.1016/j.arcmed.2024.103043

[10] Amini-Salehi E Hassanipour S Joukar F . Risk factors of non-alcoholic fatty liver disease in the Iranian adult population: a systematic review and meta-analysis. Hepat Mon 2023;23:e131523. 10.18502/ijph.v52i8.13399 PMC10512128 37744533

[11] Townsend N Kazakiewicz D Lucy Wright F . Epidemiology of cardiovascular disease in Europe. Nat Rev Cardiol 2022;19:133–143. 34497402 10.1038/s41569-021-00607-3

[12] Tsao CW Aday AW Almarzooq ZI . Heart disease and stroke statistics-2023 update: a report from the American Heart Association. Circulation 2023;147:e93–e621. 36695182 10.1161/CIR.0000000000001123 PMC12135016

[13] Conrad N Molenberghs G Verbeke G . Trends in cardiovascular disease incidence among 22 million people in the UK over 20 years: population based study. BMJ 2024;385:e078523. 38925788 10.1136/bmj-2023-078523 PMC11203392

[14] Naghipour A Amini-Salehi E Orang Gorabzarmakhi M . Effects of gut microbial therapy on lipid profile in individuals with non-alcoholic fatty liver disease: an umbrella meta-analysis study. Syst Rev 2023;12:144. 37605283 10.1186/s13643-023-02299-x PMC10441764

[15] Roth GA Mensah GA Johnson CO . Global burden of cardiovascular diseases and risk factors, 1990-2019: update from the GBD 2019 study. J Am Coll Cardiol 2020;76:2982–3021. 33309175 10.1016/j.jacc.2020.11.010 PMC7755038

[16] Godugu S Sinha T Pradeepan M . Unraveling The Enigma Of Aortic Dissection: From Genetics To Innovative Therapies. Cureus 2024;16:e57803. 38721226 10.7759/cureus.57803 PMC11077317

[17] Kumar S Khatri M Kumar S . Comparative efficacy and safety profiles of high-power, short-duration and low-power, long-duration radiofrequency ablation in atrial fibrillation: a systematic review and meta-analysis. J Innov Card Rhythm Manag 2023;14:5514–5527. 37492695 10.19102/icrm.2023.14072 PMC10364668

[18] Khatri M Kumar S Mahfooz K . Clinical outcomes of polymer-free versus polymer-coated drug-eluting stents in patients with coronary artery disease: a systematic review and meta-analysis. Cureus 2023;15:e38215. 37252538 10.7759/cureus.38215 PMC10224769

[19] Islam R Islam H . Comment on: Long-term outcomes in patients with acute myocardial infarction and no ischemic changes on electrocardiogram. Heart Lung 2023;60:154–155. 36878810 10.1016/j.hrtlng.2023.02.013

[20] Ahmed D Islam H Reyaz I . Evolving burden of peripheral artery disease attributable to smoking in 38 Oecd countries from 1990-2019: global, regional, national variations, age dynamics, and implications for global health. Arterioscler Thromb Vasc Biol 2024;44(Suppl_1):A2025.

[21] Vakilpour A Amini-Salehi E Soltani Moghadam A . The effects of gut microbiome manipulation on glycemic indices in patients with non-alcoholic fatty liver disease: a comprehensive umbrella review. Nutr Diabetes 2024;14:25. 38729941 10.1038/s41387-024-00281-7 PMC11087547

[22] Jyotsna F Ahmed A Kumar K . Exploring the complex connection between diabetes and cardiovascular disease: analyzing approaches to mitigate cardiovascular risk in patients with diabetes. Cureus 2023;15:e43882. 37746454 10.7759/cureus.43882 PMC10511351

[23] Akil L Ahmad HA . Relationships between obesity and cardiovascular diseases in four southern states and Colorado. J Health Care Poor Underserved 2011;22(4 Suppl):61–72. 22102306 10.1353/hpu.2011.0166 PMC3250069

[24] Hlyan NP Arif T Jaufar SS . From sugar spikes to pressure peaks: navigating the world of diabetes, hypertension, obesity, and kidney health. Cureus 2024;16:e57241. 38686257 10.7759/cureus.57241 PMC11056813

[25] Vemparala P Islam R Vanodia M . Assessing the global burden and trends of stroke attributable to smoking in 38 OECD countries from 1990 to 2019: a benchmarking analysis. Arterioscler Thromb Vasc Biol 2024;44(Suppl_1):A2026.

[26] Markandu K Dekhne A Islam H . 1420-P: global burden and trend of type 2 dm in 38 OECD countries from 1990–2019—a benchmarking systematic analysis. Diabetes 2024;73:1420-P.

[27] Cheemala SC Syed S Bibi R . Unraveling the gut microbiota: key insights into its role in gastrointestinal and cardiovascular health. J Adv Med Med Res 2024;36:34–47.

[28] Amini-Salehi E Mahapatro A Korsapati RR . Exploring the relationship between gut microbiome modulation and blood pressure in type 2 diabetes: an umbrella review. Nutr Metab Cardiovasc Dis 2024;34:2046–2054. 38902190 10.1016/j.numecd.2024.05.017

[29] Parab P Chaudhary P Mukhtar S . Role of glucagon-like peptide-1 (GLP-1) receptor agonists in cardiovascular risk management in patients with type 2 diabetes mellitus: a systematic review. Cureus 2023;15:e45487. 37859909 10.7759/cureus.45487 PMC10584355

[30] Marx N Husain M Lehrke M . GLP-1 receptor agonists for the reduction of atherosclerotic cardiovascular risk in patients with type 2 diabetes. Circulation 2022;146:1882–1894. 36508493 10.1161/CIRCULATIONAHA.122.059595

[31] Sheahan KH Wahlberg EA Gilbert MP . An overview of GLP-1 agonists and recent cardiovascular outcomes trials. Postgrad Med J 2020;96:156–161. 31801807 10.1136/postgradmedj-2019-137186 PMC7042958

[32] Saraiva FK Sposito AC . Cardiovascular effects of glucagon-like peptide 1 (GLP-1) receptor agonists. Cardiovasc Diabetol 2014;13:142. 25338737 10.1186/s12933-014-0142-7 PMC4216654

[33] Islam H Puttagunta SM Islam R . Risk of stroke with mitral stenosis: the underlying mechanism, treatment, and prevention. Cureus 2022;14:e23784. 35518523 10.7759/cureus.23784 PMC9063730

[34] Watanabe JH Kwon J Nan B . Trends in glucagon-like peptide 1 receptor agonist use, 2014 to 2022. J Am Pharm Assoc (2003) 2024;64:133–138. 37821008 10.1016/j.japh.2023.10.002

[35] Benomar L Elferjani R Hamilton J . Bibliometric analysis of the structure and evolution of research on assisted migration. Curr Forestry Rep 2022;8:199–213.

[36] Ma D Guan B Song L . A bibliometric analysis of exosomes in cardiovascular diseases from 2001 to 2021. Front Cardiovasc Med 2021;8:734514. 34513962 10.3389/fcvm.2021.734514 PMC8424118

[37] Ma C Su H Li H . Global research trends on prostate diseases and erectile dysfunction: a bibliometric and visualized study. Front Oncol 2020;10:627891. 33643922 10.3389/fonc.2020.627891 PMC7908828

[38] Zhang J Zhang Y Hu L . Global trends and performances of magnetic resonance imaging studies on acupuncture: a bibliometric analysis. Front Neurosci 2020;14:620555. 33551731 10.3389/fnins.2020.620555 PMC7854454

[39] Wan Y Shen J Ouyang J . Bibliometric and visual analysis of neutrophil extracellular traps from 2004 to 2022. Front Immunol 2022;13:1025861. 36341351 10.3389/fimmu.2022.1025861 PMC9634160

[40] Meng T Wang P Ding J . Global research trends on ventricular remodeling: a bibliometric analysis from 2012 to 2022. Curr Probl Cardiol 2022;47:101332. 35870550 10.1016/j.cpcardiol.2022.101332

[41] Jiang J Lyu W Chen N . A bibliometric analysis of diffuse large B-cell lymphoma research from 2001 to 2020. Comput Biol Med 2022;146:105565. 35594683 10.1016/j.compbiomed.2022.105565

[42] van Eck NJ Waltman L . Software survey: VOSviewer, a computer program for bibliometric mapping. Scientometrics 2010;84:523–538. 20585380 10.1007/s11192-009-0146-3 PMC2883932

[43] Chen C . CiteSpace II: detecting and visualizing emerging trends and transient patterns in scientific literature. J Am Soc Inf Sci Technol 2006;57:359–377.

[44] Synnestvedt MB Chen C Holmes JH . CiteSpace II: visualization and knowledge discovery in bibliographic databases. AMIA Annu Symp Proc 2005;2005:724–728. 16779135 PMC1560567

[45] Wei W Jiang Z . A bibliometrix-based visualization analysis of international studies on conversations of people with aphasia: Present and prospects. Heliyon 2023;9:e16839. 37346333 10.1016/j.heliyon.2023.e16839 PMC10279826

[46] Einarson TR Acs A Ludwig C . Prevalence of cardiovascular disease in type 2 diabetes: a systematic literature review of scientific evidence from across the world in 2007-2017. Cardiovasc Diabetol 2018;17:83. 29884191 10.1186/s12933-018-0728-6 PMC5994068

[47] Best JH Hoogwerf BJ Herman WH . Risk of cardiovascular disease events in patients with type 2 diabetes prescribed the glucagon-like peptide 1 (GLP-1) receptor agonist exenatide twice daily or other glucose-lowering therapies: a retrospective analysis of the LifeLink database. Diabetes Care 2011;34:90–95. 20929995 10.2337/dc10-1393 PMC3005487

[48] Islam R Ahmed M Ullah W . Effect of caffeine in hypertension. Curr Probl Cardiol 2023;48:101892. 37394201 10.1016/j.cpcardiol.2023.101892

[49] Islam H Potluri G Markandu K . 1451-P: burden of type 1 diabetes mellitus (T1DM) and its trend in G20 countries from 1990–2019—a benchmarking analysis. Diabetes 2024;73:1451-P.

[50] Maddineni K Parisapogu A Teja Chinthapalli M . 1274-P: evolving burden of stroke attributable to high fasting plasma glucose in the United States from 1990–2019—a benchmarking systematic analysis. Diabetes 2024;73:1274-P.

[51] Heuvelman VD Van Raalte DH Smits MM . Cardiovascular effects of glucagon-like peptide 1 receptor agonists: from mechanistic studies in humans to clinical outcomes. Cardiovasc Res 2020;116:916–930. 31825468 10.1093/cvr/cvz323

[52] Stewart J Manmathan G Wilkinson P . Primary prevention of cardiovascular disease: a review of contemporary guidance and literature. JRSM Cardiovasc Dis 2017;6:2048004016687211. 28286646 10.1177/2048004016687211 PMC5331469

[53] Islam H Islam R . Cardiovascular outcomes of patients referred to home based cardiac rehabilitation. Heart Lung 2023;60:146–147. 36914550 10.1016/j.hrtlng.2023.02.015

[54] Drucker DJ Buse JB Taylor K . Exenatide once weekly versus twice daily for the treatment of type 2 diabetes: a randomised, open-label, non-inferiority study. Lancet 2008;372:1240–1250. 18782641 10.1016/S0140-6736(08)61206-4

[55] Buse JB Rosenstock J Sesti G . Liraglutide once a day versus exenatide twice a day for type 2 diabetes: a 26-week randomised, parallel-group, multinational, open-label trial (LEAD-6). Lancet 2009;374:39–47. 19515413 10.1016/S0140-6736(09)60659-0

[56] Marre M Shaw J Brändle M . Liraglutide, a once-daily human GLP-1 analogue, added to a sulphonylurea over 26 weeks produces greater improvements in glycaemic and weight control compared with adding rosiglitazone or placebo in subjects with Type 2 diabetes (LEAD-1 SU). Diabet Med 2009;26:268–278. 19317822 10.1111/j.1464-5491.2009.02666.x PMC2871176

[57] Nauck M Frid A Hermansen K . Long-term efficacy and safety comparison of liraglutide, glimepiride and placebo, all in combination with metformin in type 2 diabetes: 2-year results from the LEAD-2 study. Diabetes Obes Metab 2013;15:204–212. 22985213 10.1111/dom.12012

[58] Garber A Henry R Ratner R . Liraglutide versus glimepiride monotherapy for type 2 diabetes (LEAD-3 Mono): a randomised, 52-week, phase III, double-blind, parallel-treatment trial. Lancet 2009;373:473–481. 18819705 10.1016/S0140-6736(08)61246-5

[59] Zinman B Gerich J Buse JB . Efficacy and safety of the human glucagon-like peptide-1 analog liraglutide in combination with metformin and thiazolidinedione in patients with type 2 diabetes (LEAD-4 Met+TZD). Diabetes Care 2009;32:1224–1230. 19289857 10.2337/dc08-2124 PMC2699702

[60] Russell-Jones D Vaag A Schmitz O . Liraglutide vs insulin glargine and placebo in combination with metformin and sulfonylurea therapy in type 2 diabetes mellitus (LEAD-5 met+SU): a randomised controlled trial. Diabetologia 2009;52:2046–2055. 19688338 10.1007/s00125-009-1472-y PMC2744824

[61] Blevins T Pullman J Malloy J . DURATION-5: exenatide once weekly resulted in greater improvements in glycemic control compared with exenatide twice daily in patients with type 2 diabetes. J Clin Endocrinol Metab 2011;96:1301–1310. 21307137 10.1210/jc.2010-2081

[62] Smits MM Tonneijck L Muskiet MH . GLP-1-based therapies have no microvascular effects in type 2 diabetes mellitus: an acute and 12-week randomized, double-blind, placebo-controlled trial. Arterioscler Thromb Vasc Biol 2016;36:2125–2132. 27562916 10.1161/ATVBAHA.116.307930

[63] Smits MM Muskiet MH Tonneijck L . GLP-1 receptor agonist exenatide increases capillary perfusion independent of nitric oxide in healthy overweight men. Arterioscler Thromb Vasc Biol 2015;35:1538–1543. 25908765 10.1161/ATVBAHA.115.305447

[64] Holman RR Bethel MA Mentz RJ . Effects of once-weekly exenatide on cardiovascular outcomes in type 2 diabetes. N Engl J Med 2017;377:1228–1239. 28910237 10.1056/NEJMoa1612917 PMC9792409

[65] Cuthbertson DJ Irwin A Gardner CJ . Improved glycaemia correlates with liver fat reduction in obese, type 2 diabetes, patients given glucagon-like peptide-1 (GLP-1) receptor agonists. PLoS One 2012;7:e50117. 23236362 10.1371/journal.pone.0050117 PMC3516516

[66] Marso SP Bain SC Consoli A . Semaglutide and cardiovascular outcomes in patients with type 2 diabetes. N Engl J Med 2016;375:1834–1844. 27633186 10.1056/NEJMoa1607141

[67] Marso SP Daniels GH Brown-Frandsen K . Liraglutide and cardiovascular outcomes in type 2 diabetes. N Engl J Med 2016;375:311–322. 27295427 10.1056/NEJMoa1603827 PMC4985288

[68] Pfeffer MA Claggett B Diaz R . Lixisenatide in patients with type 2 diabetes and acute coronary syndrome. N Engl J Med 2015;373:2247–2257. 26630143 10.1056/NEJMoa1509225

[69] Krishnan A Schneider CV Hadi Y . Cardiovascular and mortality outcomes with GLP-1 receptor agonists vs other glucose-lowering drugs in individuals with NAFLD and type 2 diabetes: a large population-based matched cohort study. Diabetologia 2024;67:483–493. 38117293 10.1007/s00125-023-06057-5 PMC10844347

[70] Yen FS Hou MC Wei JC . Effects of glucagon-like peptide-1 receptor agonists on liver-related and cardiovascular mortality in patients with type 2 diabetes. BMC Med 2024;22:8. 38172833 10.1186/s12916-023-03228-4 PMC10765623

[71] Margulies KB Hernandez AF Redfield MM . Effects of liraglutide on clinical stability among patients with advanced heart failure and reduced ejection fraction: a randomized clinical trial. JAMA 2016;316:500–508. 27483064 10.1001/jama.2016.10260 PMC5021525

[72] Henein MY Vancheri S Longo G . The role of inflammation in cardiovascular disease. Int J Mol Sci 2022;23:12906. 36361701 10.3390/ijms232112906 PMC9658900

[73] Ma Y Bhuiyan MS Kim I . Editorial: metabolic regulation in the development of cardiovascular diseases. Front Cell Dev Biol 2021;9:768689. 34722552 10.3389/fcell.2021.768689 PMC8548466

[74] Kosmas CE Bousvarou MD Kostara CE . Insulin resistance and cardiovascular disease. J Int Med Res 2023;51:3000605231164548. 36994866 10.1177/03000605231164548 PMC10069006

[75] Ussher JR Drucker DJ . Glucagon-like peptide 1 receptor agonists: cardiovascular benefits and mechanisms of action. Nat Rev Cardiol 2023;20:463–474. 36977782 10.1038/s41569-023-00849-3

[76] Mentz RJ Bethel MA Gustavson S . Baseline characteristics of patients enrolled in the Exenatide Study of Cardiovascular Event Lowering (EXSCEL). Am Heart J 2017;187:1–9. 28454792 10.1016/j.ahj.2017.02.005 PMC9849915

[77] Marx N Davies MJ Grant PJ . Guideline recommendations and the positioning of newer drugs in type 2 diabetes care. Lancet Diabetes Endocrinol 2021;9:46–52. 33159841 10.1016/S2213-8587(20)30343-0 PMC12140926

[78] Bethel MA Patel RA Merrill P . Cardiovascular outcomes with glucagon-like peptide-1 receptor agonists in patients with type 2 diabetes: a meta-analysis. Lancet Diabetes Endocrinol 2018;6:105–113. 29221659 10.1016/S2213-8587(17)30412-6

[79] McMurray JJ Holman RR Haffner SM . Effect of valsartan on the incidence of diabetes and cardiovascular events. N Engl J Med 2010;362:1477–1490. 20228403 10.1056/NEJMoa1001121

[80] Cheng AY Leiter LA . Glucose lowering and cardiovascular disease: what do we know and what should we do? Eur J Cardiovasc Prev Rehabil 2010;17(Suppl 1):S25–S31. 20489417 10.1097/01.hjr.0000368194.32356.5f

[81] Chew EY Ambrosius WT Davis MD . Effects of medical therapies on retinopathy progression in type 2 diabetes. N Engl J Med 2010;363:233–244. 20587587 10.1056/NEJMoa1001288 PMC4026164

[82] Gerstein HC Colhoun HM Dagenais GR . Dulaglutide and cardiovascular outcomes in type 2 diabetes (REWIND): a double-blind, randomised placebo-controlled trial. Lancet 2019;394:121–130. 31189511 10.1016/S0140-6736(19)31149-3

[83] Green JB Hernandez AF D’Agostino RB . Harmony outcomes: a randomized, double-blind, placebo-controlled trial of the effect of albiglutide on major cardiovascular events in patients with type 2 diabetes mellitus-Rationale, design, and baseline characteristics. Am Heart J 2018;203:30–38. 30015066 10.1016/j.ahj.2018.03.030

[84] Scirica BM Bhatt DL Braunwald E . Saxagliptin and cardiovascular outcomes in patients with type 2 diabetes mellitus. N Engl J Med 2013;369:1317–1326. 23992601 10.1056/NEJMoa1307684

[85] Neal B Perkovic V Mahaffey KW . Canagliflozin and cardiovascular and renal events in type 2 diabetes. N Engl J Med 2017;377:644–657. 28605608 10.1056/NEJMoa1611925

[86] Garvey WT Frias JP Jastreboff AM . Tirzepatide once weekly for the treatment of obesity in people with type 2 diabetes (SURMOUNT-2): a double-blind, randomised, multicentre, placebo-controlled, phase 3 trial. Lancet 2023;402:613–626. 37385275 10.1016/S0140-6736(23)01200-X

[87] Garvey WT Batterham RL Bhatta M . Two-year effects of semaglutide in adults with overweight or obesity: the STEP 5 trial. Nat Med 2022;28:2083–2091. 36216945 10.1038/s41591-022-02026-4 PMC9556320

[88] Tsatsoulis A Paschou SA . Metabolically healthy obesity: criteria, epidemiology, controversies, and consequences. Curr Obes Rep 2020;9:109–120. 32301039 10.1007/s13679-020-00375-0

[89] Copur S Tanriover C Yavuz F . Tirzepatide and potential use for metabolically healthy obesity. Eur J Intern Med 2023;113:1–5. 37183081 10.1016/j.ejim.2023.05.012

[90] Shen Y Cai R Sun J . Diabetes mellitus as a risk factor for incident chronic kidney disease and end-stage renal disease in women compared with men: a systematic review and meta-analysis. Endocrine 2017;55:66–76. 27477292 10.1007/s12020-016-1014-6

[91] Siddiqui K George TP Joy SS . Risk factors of chronic kidney disease among type 2 diabetic patients with longer duration of diabetes. Front Endocrinol 2022;13:1079725. 10.3389/fendo.2022.1079725 PMC9780388 36568108

[92] Damtie S Biadgo B Baynes HW . Chronic kidney disease and associated risk factors assessment among diabetes mellitus patients at a Tertiary Hospital, Northwest Ethiopia. Ethiop J Health Sci 2018;28:691–700. 30607085 10.4314/ejhs.v28i6.3 PMC6308752

[93] Michos ED Bakris GL Rodbard HW . Glucagon-like peptide-1 receptor agonists in diabetic kidney disease: a review of their kidney and heart protection. Am J Prev Cardiol 2023;14:100502. 37313358 10.1016/j.ajpc.2023.100502 PMC10258236

[94] Yu JH Park SY Lee DY . GLP-1 receptor agonists in diabetic kidney disease: current evidence and future directions. Kidney Res Clin Pract 2022;41:136–149. 35391537 10.23876/j.krcp.22.001 PMC8995488

[95] Lin Y Wang T-H Tsai M-L . The cardiovascular and renal effects of glucagon-like peptide 1 receptor agonists in patients with advanced diabetic kidney disease. Cardiovasc Diabetol 2023;22:60. 36932379 10.1186/s12933-023-01793-9 PMC10024371

[96] Kawanami D Takashi Y . GLP-1 receptor agonists in diabetic kidney disease: from clinical outcomes to mechanisms. Front Pharmacol 2020;11:967. 32694999 10.3389/fphar.2020.00967 PMC7338581

[97] Alani H Tamimi A Tamimi N . Cardiovascular co-morbidity in chronic kidney disease: current knowledge and future research needs. World J Nephrol 2014;3:156–168. 25374809 10.5527/wjn.v3.i4.156 PMC4220348

[98] Mann JFE Ørsted DD Brown-Frandsen K . Liraglutide and renal outcomes in type 2 diabetes. N Engl J Med 2017;377:839–848. 28854085 10.1056/NEJMoa1616011

[99] Muskiet MHA Tonneijck L Huang Y . Lixisenatide and renal outcomes in patients with type 2 diabetes and acute coronary syndrome: an exploratory analysis of the ELIXA randomised, placebo-controlled trial. Lancet Diabetes Endocrinol 2018;6:859–869. 30292589 10.1016/S2213-8587(18)30268-7

[100] Cai X She M Xu M . GLP-1 treatment protects endothelial cells from oxidative stress-induced autophagy and endothelial dysfunction. Int J Biol Sci 2018;14:1696–1708. 30416384 10.7150/ijbs.27774 PMC6216037

[101] Tanaka T Higashijima Y Wada T . The potential for renoprotection with incretin-based drugs. Kidney Int 2014;86:701–711. 25007170 10.1038/ki.2014.236

[102] Górriz JL Soler MJ Navarro-González JF . GLP-1 receptor agonists and diabetic kidney disease: a call of attention to nephrologists. J Clin Med 2020;9:947. 32235471 10.3390/jcm9040947 PMC7231090

[103] Dagli N Kumar S Ahmad R . An update on semaglutide research: a bibliometric analysis and a literature review. Cureus 2023;15:e46510. 37808605 10.7759/cureus.46510 PMC10552354

[104] Shou X Wang Y Duan C . Knowledge domain and emerging trends of glucagon-like peptide 1 receptor agonists in cardiovascular research: a bibliometric analysis. Curr Probl Cardiol 2023;48:101194. 35395332 10.1016/j.cpcardiol.2022.101194

