# Supplementary material for: Glucagon-like peptide-1 agonists in cardiovascular diseases: a bibliometric analysis from inception to 2023
Source: Ann Med Surg (Lond). 2024 Sep 25;86(11):6602–18. doi: 10.1097/MS9.0000000000002592 (PMC11543192; doi:10.1097/MS9.0000000000002592)
Supplement: Supplementary file 1 [file ms9-86-6602-s001.docx]

**Table S1: Search strategy designed for Web of Science Database**

| ISI Search formula | ("Cardiovascular Diseases" OR "Cardiovascular Disease" OR "Disease, Cardiovascular" OR "Major Adverse Cardiac Events" OR "Cardiac Events" OR "Cardiac Event" OR "Event, Cardiac" OR "Adverse Cardiac Event" OR "Adverse Cardiac Events" OR "Cardiac Event, Adverse" OR "Cardiac Events, Adverse" OR "Cardiovascular Abnormalities" OR "Abnormalities, Cardiovascular" OR "Abnormality, Cardiovascular" OR "Cardiovascular Abnormality" OR "Heart Diseases" OR "Heart Disease" OR "Cardiac Diseases" OR "Cardiac Disease" OR "Cardiac Disorders" OR "Cardiac Disorder" OR "Heart Disorders" OR "Heart Disorder" OR "Heart Failure" OR "Cardiac Failure" OR "Heart Decompensation" OR "Decompensation, Heart" OR "Heart Failure, Right-Sided" OR "Heart Failure, Right Sided" OR "Right-Sided Heart Failure" OR "Right Sided Heart Failure" OR "Myocardial Failure" OR "Congestive Heart Failure" OR "Heart Failure, Congestive" OR "Heart Failure, Left-Sided" OR "Heart Failure, Left Sided" OR "Left-Sided Heart Failure" OR "Left Sided Heart Failure" OR "Hypertension" OR "Blood Pressure, High" OR "Blood Pressures, High" OR "High Blood Pressure" OR "High Blood Pressures" OR "Myocardial Ischemia" OR "Ischemia, Myocardial" OR "Ischemias, Myocardial" OR "Myocardial Ischemias" OR "Ischemic Heart Disease" OR "Heart Disease, Ischemic" OR "Disease, Ischemic Heart" OR "Diseases, Ischemic Heart" OR "Heart Diseases, Ischemic" OR "Ischemic Heart Diseases" OR "Atherosclerosis" OR "Atheroscleroses" OR "Atherogenesis" OR "Atherogeneses") AND ("Exenatide" OR "Byetta" OR "AC 2993" OR "Bydureon" OR "Exendin-4" OR "Ex4 Peptide" OR "Peptide, Ex4" OR "Exendin 4" OR "ITCA 650" OR "AC 2993 LAR" OR "Liraglutide" OR "Victoza" OR "Saxenda" OR "NN 2211" OR "NN2211" OR "NN-2211" OR "dulaglutide" OR "LY 2189265" OR "LY-2189265" OR "LY2189265" OR "Trulicity" OR "semaglutide" OR "rybelsus" OR "Wegovy" OR "Ozempic" OR "rGLP-1 protein" OR "Eperzan" OR "Tanzeum" OR "albiglutide" OR "lixisenatide" OR "DES-38-proline-exendine-4 (Heloderma suspectum)-(1-39)-peptidylpenta-l-lysyl-l-lysinamide" OR "Adlyxin" OR "AQVE-10010" OR "ZP10A peptide" OR "ZP 10" OR "ZP-10" OR "Lyxumia" OR "AVE 010" OR "AVE-010" OR "AVE 0010" OR "AVE0010" OR "AVE-0010" OR "CNTO 736" OR "CNTO-736" OR "CNTO736" OR "BMS 686117" OR "BMS686117" OR "BMS-686117" OR "DA-JC4" OR "tirzepatide" OR "zepbound" OR "LY3298176" OR "DA5-CH peptide" OR "ZP2495" OR "E2HSA protein" OR "polyethylene glycol loxenatide" OR "WB4-24") |
| --- | --- |
